# Assessing Transcriptomic Responses to Oxidative Stress: Contrasting Wild-Type Arabidopsis Seedlings with *dss1(I)* and *dss1(V)* Gene Knockout Mutants

**DOI:** 10.3390/ijms25126291

**Published:** 2024-06-07

**Authors:** Ivana Nikolić, Mira Milisavljević, Gordana Timotijević

**Affiliations:** Group for Plant Molecular Biology, Institute of Molecular Genetics and Genetic Engineering, University of Belgrade, Vojvode Stepe 444a, 11042 Belgrade, Serbia; ivana.nikolic@imgge.bg.ac.rs (I.N.); milisavljevicm@imgge.bg.ac.rs (M.M.)

**Keywords:** RNA-seq, AtDSS1, oxidative stress, homologous recombination, CRISPR/Cas9 plant mutants, *Arabidopsis thaliana*, *Ustilago maydis*

## Abstract

Oxidative stress represents a critical facet of the array of abiotic stresses affecting crop growth and yield. In this paper, we investigated the potential differences in the functions of two highly homologous Arabidopsis DSS1 proteins in terms of maintaining genome integrity and response to oxidative stress. In the context of homologous recombination (HR), it was shown that overexpressing AtDSS1(I) using a functional complementation test increases the resistance of the Δ*dss1* mutant of *Ustilago maydis* to genotoxic agents. This indicates its conserved role in DNA repair via HR. To investigate the global transcriptome changes occurring in *dss1* plant mutant lines, gene expression analysis was conducted using Illumina RNA sequencing technology. Individual RNA libraries were constructed from three total RNA samples isolated from *dss1(I)*, *dss1(V)*, and wild-type (WT) plants under hydrogen peroxide-induced stress. RNA-Seq data analysis and real-time PCR identification revealed major changes in gene expression between mutant lines and WT, while the *dss1(I)* and *dss1(V)* mutant lines exhibited analogous transcription profiles. The Kyoto Encyclopedia of Genes and Genomes enrichment analysis revealed significantly enriched metabolic pathways. Notably, genes associated with HR were upregulated in *dss1* mutants compared to the WT. Otherwise, genes of the metabolic pathway responsible for the synthesis of secondary metabolites were downregulated in both *dss1* mutant lines. These findings highlight the importance of understanding the molecular mechanisms of plant responses to oxidative stress.

## 1. Introduction

All living organisms are exposed to unfavorable stimuli for shorter or longer periods of time. Unlike animals, plants are stationary life forms that cannot escape stressful factors by moving away. Nonetheless, plants successfully survive because they have developed numerous defense mechanisms and tolerance to environmental stresses [1]. Any harmful biotic or abiotic factor has the potential to induce oxidative stress in plant cells [2]. Conditions for oxidative stress occur when the cell enters a state of imbalance between the production of reactive oxygen species (ROS) and its ability to detoxify these reactive intermediates. Highly reactive molecules can cause serious damage to biomolecules such as proteins, lipids, and DNA, leading to a disturbance in cellular functions and even cell death [3]. Plants respond to oxidative stress through a complex antioxidant defense system that includes both enzymatic and non-enzymatic mechanisms. Superoxide dismutase, catalase, and ascorbate peroxidase are enzymatic antioxidants responsible for transforming ROS into less harmful molecules [4]. Non-enzymatic antioxidants are different compounds, such as ascorbate, glutathione, and tocopherol, that have the ability to neutralize ROS [3]. At the foundation of plant defense against oxidative stress lie intricate signaling networks. These networks are pivotal in regulating the production of defensive secondary metabolites and coordinating alterations in gene expression. Understanding these mechanisms is important for developing strategies to enhance plant resistance to stress. New insights can enhance crop yields and overcome agriculture issues arising from climate change.

Along with the well-known components of the antioxidant system, members of a large family of naturally disordered proteins (IDPs—intrinsically disordered proteins) are also essential for plants to react effectively to stress. IDPs are highly represented in the proteome of eukaryotes. Plant IDPs play a key role in plant biology, acting as a connection between different intracellular regulatory signals and signals from the external environment. Research has shown that these proteins play a significant role in abiotic stress, transcriptional regulation, light perception, and plant development [5]. Late embryogenesis abundant (LEA) proteins, gibberellic acid-insensitive repressor of ga1-3 scarecrow (GRAS) proteins, and cryptochromes (CRYs) are well-known protein families in plants that link intracellular regulatory processes with external environmental signals [5]. Among these families, LEA proteins are the most prominent and are typically induced by various environmental signals such as cold, frost, heat, drought, salinity, and increased evaporation [6]. These proteins serve diverse functions. For instance, LEA proteins can interact with partially denatured polypeptides, preventing protein aggregation in the cytoplasm during dehydration. Additionally, they can serve as both chaperones and transcription factors [7]. As transcription factors, LEA proteins connect external signals to specific gene expression patterns. One IDP, specifically the DSS1 (deletion of split hand/split foot 1) protein, could be one of the many factors involved in the plant stress response, operating within the proteasome machinery alongside various other factors. It was shown that the absence of DSS1 proteins in *Arabidopsis thaliana* mutant lines exposed to hydrogen peroxide (H_2_O_2_) leads to the accumulation of oxidized proteins and disruption of protein homeostasis [8].

DSS1 is a small, acidic, and highly conserved eukaryotic protein. It belongs to a large family of naturally disordered proteins lacking three-dimensional structure. DSS1 proteins also lack well-defined secondary elements. Even when DSS1 is bound to other molecules, large regions of the protein remain disordered, and only a limited secondary helical structure is observed at the C-terminus of the protein [9,10,11]. Despite the absence of a 3D structure, IDPs participate in cell functioning and help maintain the stability of biological complexes, primarily as regulatory components [12]. DSS1 is a multifunctional protein that participates in various biological processes through its interaction with various components of numerous protein complexes. When this flexible protein binds to partner proteins, it acquires final 3D conformation. The molecular weights of DSS1 proteins range from 7–9 kDa, with lengths of polypeptide chains ranging from 70 to 90 amino acids in different organisms. Multiple alignments of amino acid sequences of DSS1 orthologues exhibit high levels of homology between fungi, plants, animals, and humans [11]. The fully conserved region of 15 amino acids presents a functionally significant domain for interaction with other proteins [13]. The secondary structure of DSS1 contains a transient α-helix at the end of the C-terminus and two conserved acidic regions [13,14].

One of the first well-defined roles of DSS1 is its involvement in the regulation of homologous recombination (HR), the most precise mechanism of DNA double-strand break repair [15]. In most eukaryotes, the core of HR machinery is comprised of recombinase RAD51, its mediator BRCA2, and regulator DSS1. The occurrence of DNA double-strand breaks induces the recruitment of a number of proteins that process the breaks, generating long 3′ DNA tails that are then coated with RPA homofilament. Recombinase RAD51 uses this single-stranded DNA to search for and invade homologous DNA. However, displacement of RPA by RAD51 requires the BRCA2 protein, which is conserved in higher eukaryotes and also found in fungi from the Basidiomycota division, such as *Ustilago maydis* [16]. The interplay of DSS1 and BRCA2 is essential for the properly regulated HR activity of the BRCA2 machinery, contributing to the maintenance of genome integrity [13,14,15,16,17,18,19,20,21].

The suppressor of exocyst mutation 1, Sem1 (or Dss1), has been hypothesized to take part in two structurally related complexes with distinct functions in mRNA processing in yeast. One of these is the ternary complex Thp3-Csn12-Sem1, which regulates transcription and facilitates pre-mRNA splicing [14,22,23,24]. In another larger complex in which Sem1/Dss1 contributes to the assembly of the nuclear pore (NCP)—transcription-export complex-2 (TREX-2), it plays a role in mRNA export.

As the smallest regulatory and structural subunit of the 26S proteasome, besides ubiquitin-binding activity, Dss1 also participates in the correct proteasome assembly. Dss1 contributes to the stability of the proteasome by effectively recruiting its subunits Rpn3 and Rpn7, which normally have a low affinity for each other [23,25,26]. Although complete assembly of the proteasome is possible in the absence of Dss1, the proteolytic activity of this proteasome is incomplete. During proteolysis, Dss1 is necessary for the fine modulation of Rpn7 in the process of efficient ATP-dependent substrate unwinding [25].

Our studies have revealed that the *A. thaliana dss1(V)* mutant has increased sensitivity to oxidative stress when compared to *dss1(I)* or wild-type (WT) plants. The lack of DSS1(V) leads to an excessive accumulation of oxidized proteins, likely caused by the impaired functioning of the 26S proteasome. Conversely, the absence of DSS1(I) in *dss1(I)* mutant plants caused only moderate accumulation of damaged proteins under H_2_O_2_-induced oxidative stress [8].

In this paper, we further explored the potential differences in the functions of two highly homologous DSS1 proteins using knockdown *dss1* mutant plants. Transcriptome analysis revealed that the absence of either *DSS1* gene caused alterations in the expression of different genes under stress conditions. In terms of transcriptional regulation, major differences between the *DSS1(I)* and *DSS1(V)* genes were not detected. However, it is important to note that the lack of significant differences does not rule out subtle variations in their regulatory functions or interactions with other cellular components. Our results imply that DSS1s act as negative regulators of the genes involved in the synthesis of secondary metabolites that play a vital role in plant defense against stress. Based on the results of the functional complementation test, we showed that the *DSS1(I)* gene may make a more substantial contribution to the HR process. Further research on the interactions of individual plant DSS1 isoforms with other proteins involved in DNA repair or studies on their interaction with DNA regulatory sequences of genes that showed altered expression in transcriptome analysis could reveal their specific contributions to cellular processes.

## 2. Results

### 2.1. Test of Functional Complementation of the Ustilago maydis Δdss1 Mutant by Heterologous Expression of AtDSS1 Variants of A. thaliana

Given that the *A. thaliana* genome contains two variants of DSS1 (AtDSS1(I)—acc. no. AT1G64750, and AtDSS1(V)—acc. no. AT5G45010) that exhibit a high degree of similarity to each other, the first goal of this work was to determine whether the products of these genes have different functions in the cell. DSS1 is highly conserved among species and acts as a regulatory molecule for the proper activity of the BRCA2 complex in homologous recombination. This process is crucial for repairing double-stranded DNA lesions. The most phylogenetically conserved domain of the BRCA2 molecule is the region that contacts and binds to the DSS1 protein. To ascertain whether both DSS1 variants contribute to HR, the genes were expressed in the *Δdss1* mutant of *U. maydis*, where the gene is deleted (knockout *dss1* mutant), and their ability to compensate for *UmDss1* deficiency was monitored.

Individual open reading frames of *AtDSS1* genes were inserted into an expression vector under a strong constitutive promoter for glyceraldehyde 3-phosphate dehydrogenase (*gap*), and the *Δdss1* mutant of *U. maydis* was then transformed with the obtained constructs. Expression of these genes in transformants was confirmed by RT-PCR using *DSS1*-specific primers. *U. maydis* actin gene expression served as an endogenous control (Figure S1).

**Figure 1:**
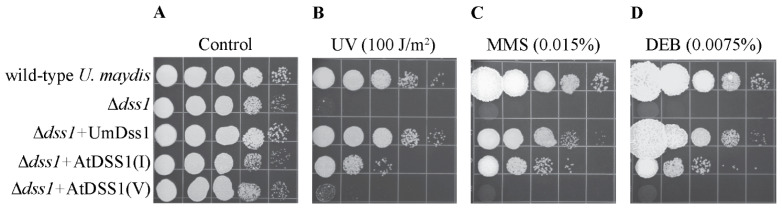
Test of functional complementation of *Ustilago maydis Δdss1* mutants. Wild strain *U. maydis*—UCM350; *Δdss1*—Dss1 mutant of *U. maydis*; *Δdss1* + UmDss1—mutant of *dss1 U. maydis* complemented by the wild-type (WT) gene *UmDss1* of *U. maydis*; *Δdss1* + AtDSS1(I)—*Δdss1* mutant of *U. maydis* complemented with WT *AtDSS1(I)* from the genome of *A. thaliana*; *Δdss1* + AtDSS1(V)—*Δdss1* mutant of *U. maydis* complemented with the WT *AtDSS1(V)* gene of *A. thaliana*. Ten µL of 10× successive serial dilutions of the cell suspension (2 × 10^7^ cells/mL) were spotted, from left to right, onto solid nutrient media at the indicated concentrations of different agents, or the Petri dish was exposed to UV radiation (**A**–**D**). Cells were grown for two days at 30 °C. The testing was performed three times and is representative of the presented results.

The *Δdss1* mutant is extremely sensitive to genotoxic agents that cause different types of lesions, such as UV radiation-induced DNA single-strand breaks, methyl methanesulfonate (MMS) acting as an alkylating agent, and diepoxybutane (DEB) acting as a cross-linking agent (Figure 1). These lesions can be repaired in different ways, and HR plays a crucial role in their repair [27]. The degree of complementation of the lack of UmDss1 in *U. maydis* by AtDSS1 proteins of *A. thaliana* was determined semi-quantitatively by comparing the survival of the transformants in the presence of genotoxic agents with the survival of the *Δdss1* mutant. The WT strain that is resistant to the tested agents, as well as the *Δdss1* mutant, in which the WT allele of *UmDss1* from *U. maydis* is overexpressed, were used as control strains. Doses of genotoxic agents that do not lead to a drop in the viability of the wild strain of *U. maydis* were used. As can be seen in Figure 1, overexpression of AtDSS1(I) causes a significant increase in the resistance of the *Δdss1* mutant when exposed to genotoxic agents. The survival rate is 3 logs (1000 times) higher compared to the mutant strain, but it is lower compared to the degree of survival achieved by homologous expression of the *UmDss1* gene. Unlike AtDSS1(I), AtDSS1(V) cannot complement Dss1 deficiency, i.e., AtDSS1(V)-transformants show the same level of sensitivity to genotoxic agents as the untransformed *Δdss1*. Remarkably, despite the significant sequence similarity (84% of identical nucleotides) between Arabidopsis *AtDSS1(I)* and *AtDSS1(V)* genes, AtDSS1(V) was unable to complement the UmDss1 deficiency in the *U. maydis* Δ*dss1* strain. Since *AtDSS1(I)* and *AtDSS1(V)* transcripts were detected in the corresponding *U. maydis* transformants, we assume that both isoforms are efficiently overexpressed from the strong *gap* promoter in *U. maydis* cells. A possible explanation for the absence of complementation by AtDSS1(V) is that the eight amino acid difference in AtDSS1(V) may affect its interaction with the *U. maydis* BRCA2 protein (named Brh2), which is a crucial interaction for the proper HR-dependent repair of DNA damages caused by the applied genotoxic agents. Also, there is a possibility that slight differences in codon usage bias can significantly affect protein expression in heterologous systems, causing the impossibility of *AtDSS1(V)* translation in *U. maydis*.

### 2.2. Transcriptome Analysis of dss1(I) and dss1(V) Mutant Lines of Arabidopsis thaliana Exposed to Oxidative Stress Induced by Hydrogen Peroxide

The complementation test confirms the results observed in knockout Arabidopsis *dss1(I)* and *dss1(V)* lines, highlighting differences in their development and oxidative stress response [8]. Transcriptome analysis was performed to clarify the molecular components underlying these differences and the possible functional significance of the previously detected differences between the two Arabidopsis *dss1* mutant lines [8].

#### 2.2.1. RNA Sequencing, Mapping, and Identification of Differentially Expressed Genes (DEGs)

A new generation technique of RNA sequencing (RNA-seq) was used to either detect the presence or quantify mRNA levels in three different biological samples, namely WT, *dss1(I),* and *dss1(V)* Arabidopsis seedlings exposed to hydrogen peroxide. A large amount of data was collected and further analyzed using bioinformatics tools. In this study, we identified several genes whose level of transcription was significantly affected by the lack of AtDSS1 protein isoforms in knockdown mutants. Also, a comparison of *dss1(I)*, *dss1(V),* and WT transcriptomes revealed differences in the expression levels of certain genes. Differentially expressed genes (DEG) were subjected to Gene Ontologies (GO) analysis to determine their function, participation in certain biological processes, and localization of proteins that they encode. Additionally, KEGG analysis was performed.

**Figure 2:**
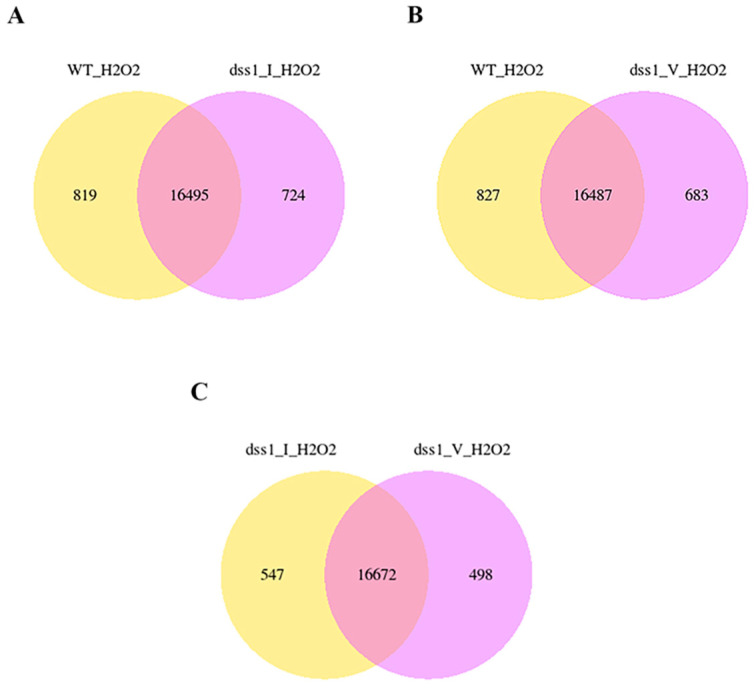
Comparison of (**A**) WT and *dss1(I)*; (**B**) WT and *dss1(V)*; and (**C**) *dss1(I)* and *dss1(V)* transcriptomes reveals the numbers of unequally and commonly expressed genes in sets of analyzed genotypes according to differentially expressed gene analysis.

The two-set Venn diagrams presented in Figure 2 visually summarize the results of the DEG analysis. The diagrams show the number of uniquely expressed genes in each genotype, as well as the number of genes co-expressed in two genotypes, shown in the intersection regions of the diagrams. Comparative analysis of WT and *dss1(I)*, WT and *dss1(V)*, as well as *dss1(I)* and *dss1(V)*, shows that many genes are co-expressed in each of the two sets of analyzed genotypes. In the diagram with the overlapping domains in Figure 2A–C, we noted that the number of co-expressed genes is almost the same, about 16,500. However, the largest number of common genes was detected in the intersection of the two *dss1* mutant lines, which is about 200 higher than between WT and *dss1(I)* or WT and *dss1(V)*.

**Figure 3:**
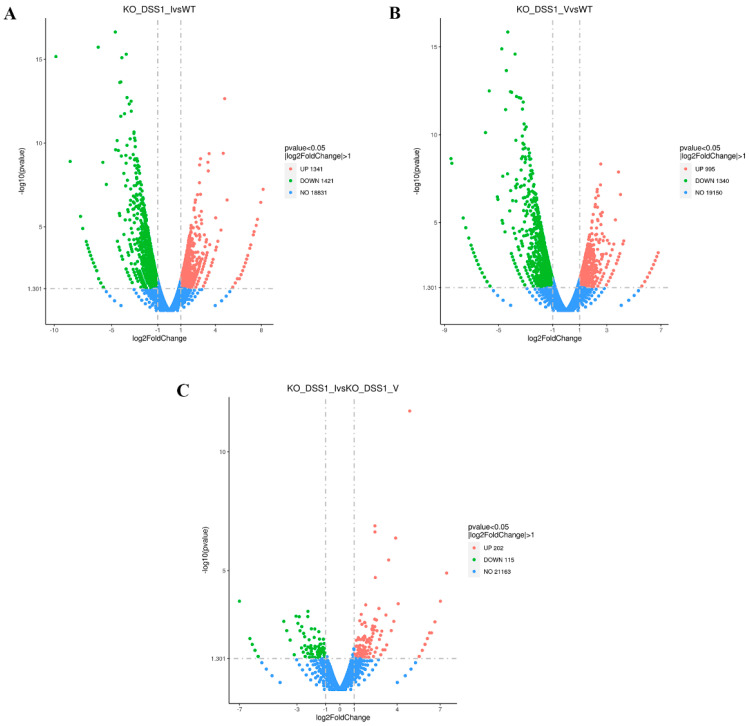
Volcano plot of all expressed transcripts in (**A**) *dss1(I)* vs. WT; (**B**) *dss1(V)* vs. WT; and (**C**) *dss1(I)* vs. *dss1(V)*. For every transcript, the fold change was plotted against the log *p*-value. Statistically significant differentially expressed genes with a fold change of ≥1.5 or ≤−1.5 are in blue; downregulated and upregulated genes are in red and green, respectively.

Biostatistical analysis was performed to identify changes in the differentially expressed genes between mutants and WT lines exposed to H_2_O_2_, and the results are shown in Volcano plots (Figure 3). In comparison to WT plants, *dss1(I)* plants showed a statistically significant increase in the expression of 1341 genes, while nearly the same number of genes (1421) had reduced expression (Figure 3A). Comparative analysis of *dss1(V)* and WT plants also showed drastic differences in gene expression: of the 76,142 expressed transcripts under oxidative stress, 2295 were differentially expressed. Specifically, 995 genes were upregulated and 1340 genes were downregulated in *dss1(V*) mutants compared to WT plants. Intercomparison of the mutant lines revealed significant but smaller differences in transcript expression: only 202 positively and 115 negatively regulated genes in *dss1(I)* compared to *dss1(V)*. The data indicate that the two *dss1* mutant lines have a similar pattern of expressed transcripts (Figure 3C).

#### 2.2.2. KEGG Pathway Enrichment Analyses of DEGs

**Figure 4:**
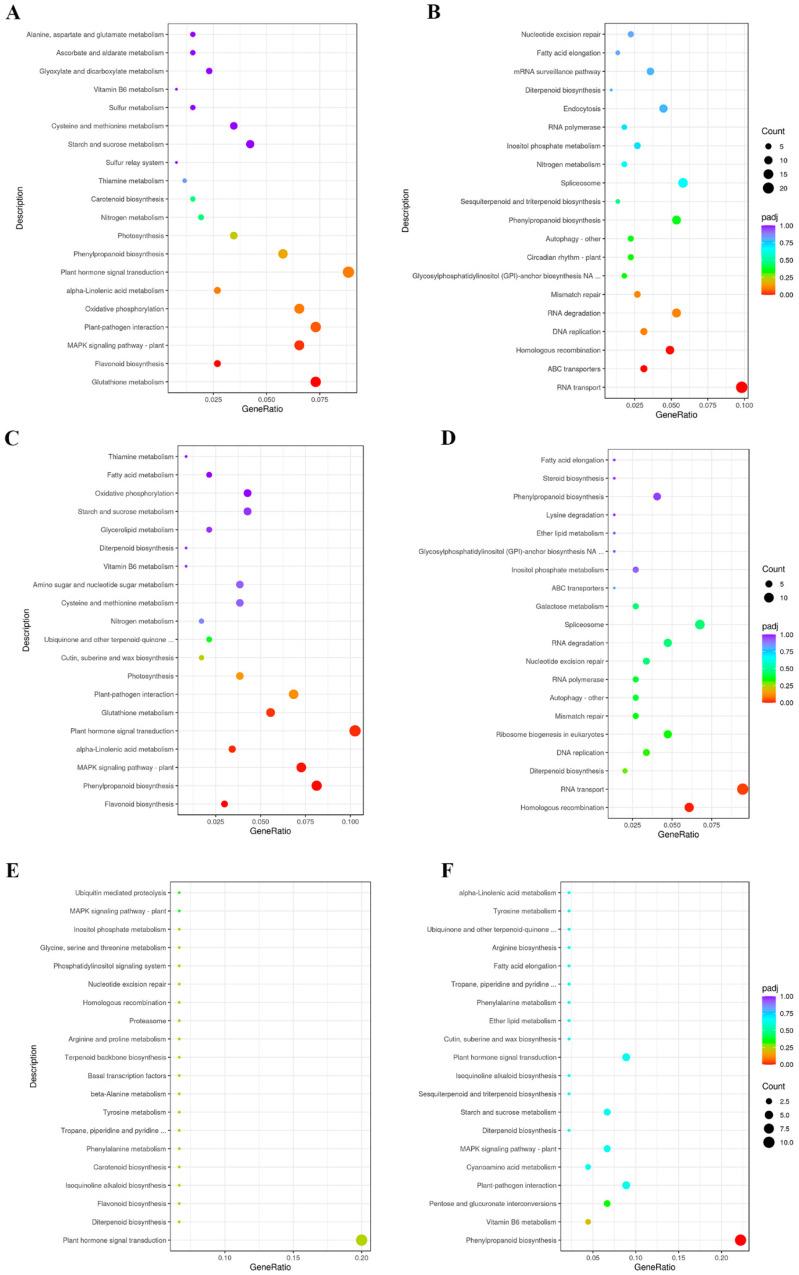
Dot plots of the results of the Kyoto Encyclopedia of Genes and Genomes (KEGG) analysis showing the most significantly up- and downregulated pathways. KEGG analysis of the (**A**) *dss1(I)* vs. WT downregulated, (**B**) *dss1(I)* vs. WT upregulated, (**C**) *dss1(V)* vs. WT downregulated, (**D**) *dss1(V)* vs. WT upregulated, (**E**) *dss1(I)* vs. *dss1(V)* downregulated, and (**F**) *dss1(I)* vs. *dss1(V)* upregulated pathways. The dot color refers to the *p*-value, and the dot size refers to the number of genes in the enrichment pathway.

Additional KEGG analysis was performed to define the functional processes involving differentially expressed genes in mutant lines and categorize them into metabolic pathways. Several pathways directly or indirectly connect with a plant’s response to abiotic stress. Genes classified in 86 KEGG pathways in *dss1(I)* had reduced expression compared to WT plants (Figure 4A and Figure S2A). Many genes involved in glutathione metabolism, flavonoid biosynthesis, and the MAPK (mitogen-activated protein kinase) signaling pathway exhibited significant changes (Figure 4A and Figure S2A). According to KEGG analysis, a significant number of genes encoding proteins that participate in 79 metabolic pathways had an elevated level of expression in the *dss1(I)* mutant line (Figure 4B and Figure S2B). Among them, we detected genes with a role in RNA transport, ABC (ATP-binding cassette) transport, and homologous recombination (Figure 4B and Figure S2B). In the case of *dss1(V)* mutants, a total of 234 negatively regulated genes were detected. These genes are associated with flavonoid biosynthesis, phenylpropanoid biosynthesis, MAPK signaling pathways, signal transduction of plant hormones, glutathione metabolism, plant-pathogen interaction, etc. (Figure 4C and Figure S2C). Regarding genes with high expression in *dss1(V)* compared to WT plants, of the 148 analyzed genes, those involved in HR and RNA transport showed statistical significance (Figure 4D and Figure S2D). KEGG analysis of differential gene expression within *dss1* mutants (*dss1(I)* vs. *dss1(V)*) exhibited no statistically significant decrease in gene expression (Figure 4E and Figure S2E). However, *dss1(I)* mutants have a significant number of upregulated genes that function in the biosynthesis of phenylpropanoids in comparison to *dss1(V)* (Figure 4F and Figure S2F).

### 2.3. Gene Expression Validation

**Figure 5:**
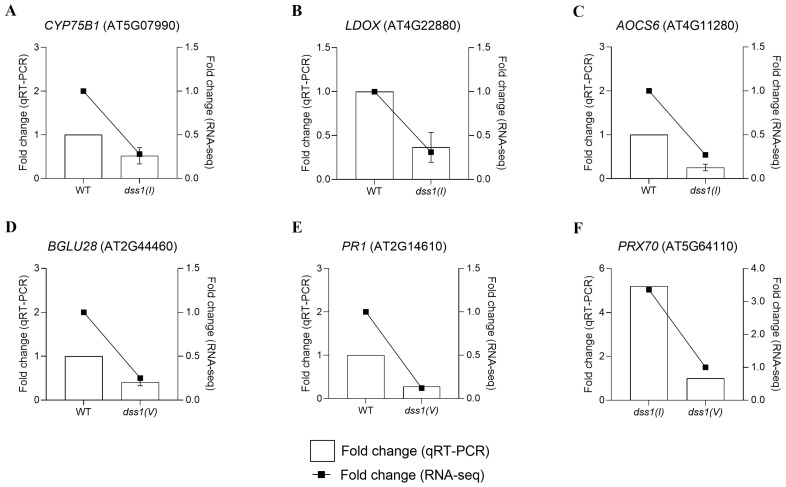
Validation of RNA-Seq results using real-time PCR (qRT-PCR). To confirm the expression profiles of the genes in the RNA-Seq results, six genes involved in the biosynthesis of secondary metabolites with decreased levels of expression in the *dss1* mutant lines were randomly selected for validation (gene accession numbers are shown in (**A**–**F**)). In the double *y*-axis figure, the left *y*-axis shows the relative expression obtained by qPCR (bar graph display), and the right *y*-axis shows the expression profiles acquired by RNA-Seq (line graph display). The bars represent the fold change obtained from qPCR, and lines marked by squares represent the fold changes obtained by RNA-seq.

**Figure 6:**
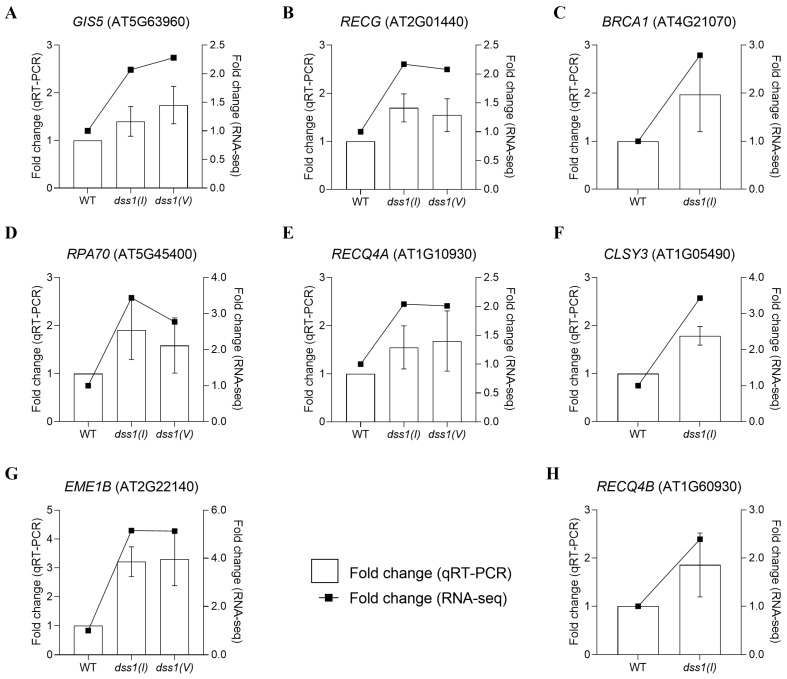
Validation of RNA-Seq results by real-time PCR (qRT-PCR). To confirm the expression profile of genes in the RNA-Seq results, nine genes involved in HR with increased levels of expression were randomly selected for validation (gene accession numbers are shown in (**A**–**H**) graphs). In the double *y*-axis figure, the left *y*-axis shows the relative expression obtained by qPCR (bar graph display), and the right *y*-axis shows the expression profiles acquired by RNA-Seq (line graph display).

In our study, we chose specific genes for further investigation and validation based on their involvement in particular biological processes (Figure S2). Previous research [8,28] highlighted the *DSS1(V)* gene’s responsiveness to stress as well as its role in defending plant cells against oxidative stress by detoxifying damaged proteins. As a result, we concentrated on processes related to secondary metabolite synthesis, given their significance in plant stress defense. We aimed to explore the correlation between the *AtDSS1* gene and other genes involved in specific processes. Another group of genes selected for further observation is associated with DNA repair and homologous recombination. Through functional complementation tests, we demonstrated that *AtDSS1(I)* could be part of this mechanism. Our primary goal is to monitor whether there is a significant correlation between the *AtDSS1* gene and other genes involved in these processes. To confirm the expression profile obtained by RNA-Seq analysis, we conducted experimental validation by real-time PCR (qRT-PCR) on genes selected based on the most significant changes in expression from *A. thaliana* seedlings exposed to H_2_O_2_ (Figure 5 and Figure 6). These genes exhibited variations in gene expression in three different genotypes: WT, *dss1(I)*, and *dss1(V)*. Six genes that were downregulated in the *dss1* lines involved in the biosynthesis pathways of different protective compounds responsible for the defense of plants against oxidative stress were chosen to validate the results of transcriptome analysis: AT5G07990 (*CYP75B1*—flavonoid 3′-monooxygenase), AT4G22880 (*LDOX*—leucoanthocyanidin dioxygenase), AT4G11280 (*AOCS6*—1-aminocyclopropane-1-carboxylate synthase 6), AT2G44460 (*BGLU28*—beta-glucosidase 28), AT2G14610 (*PR1*—pathogenesis-related gene 1), AT5G64110 (*PRX70*—peroxidase 70) (Figure 5). In both groups (WT vs. *dss1(I)* and WT vs. *dss1(V)*), our results indicate that the expression profiles of the selected genes obtained by qPCR were consistent with the expression patterns revealed by the RNA-Seq.

Additional qRT-PCR analyses were performed using the following genes involved in HR whose expression was elevated in mutant lines in comparison to the WT according to transcriptome analysis: AT5G63960 (*GIS5*—gigantea suppressor 5), AT2G01440 (*RECG*—Arabidopsis homolog of bacterial *RECG*), AT4G21070 (*BRCA1*—breast cancer susceptibility 1), AT5G45400 (*RPA70C*—replication protein A 1C), AT1G10930 (*RECQ4A—RECQ* helicase *L4A*), AT1G05490 (*CLSY*—chromatin remodeling 31), AT2G22140 (*EME1B*—essential meiotic endonuclease 1B), and AT1G60930 (*RECQ 4B—RECQ* helicase L4B) (Figure 6). The expression profiles of the chosen genes obtained through qPCR align with the expression patterns revealed by RNA-Seq.

**Figure 7:**
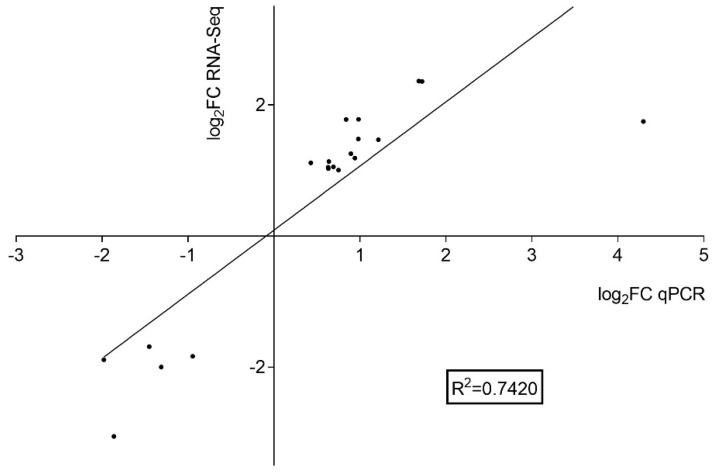
Scatter plots showing simple linear regression and the R-squared (R^2^) between the relative expression obtained by qPCR (*x*-axis) and the values obtained by RNA-seq (*y*-axis).

We also conducted a simple linear regression analysis between the relative expression results acquired through qPCR and transcriptome analysis (log_2_FC qPCR and log_2_FC RNA-Seq) (Figure 7). The calculated coefficient of determination (or R-squared) was equal to 0.7420. Our results indicate that the expression profiles of the selected genes obtained by qPCR were consistent with the expression patterns revealed by RNA-Seq.

## 3. Discussion

Research on *AtDSS1* genes points to the possibility of functional divergence between two highly homologous *DSS1* genes as regards their differential contributions to plant phenotypes and developmental dynamics [8,28]. Also, we demonstrated that *dss1(I)* and *dss1(V)* mutant plants have different sensitivities to oxidative stress compared to WT plants and to each other. This can be attributed to the different roles of DSS1(I) and DSS1(V) in maintaining protein homeostasis [8]. While DSS1 proteins exhibit high similarity, the Q44L substitution was hypothesized to induce a discernible variance between the two protein isoforms, which could also influence the observed phenotypic differences. These DSS1 proteins could have different functions in the 26S proteasome, including the elimination of damaged proteins. The absence of DSS1(V) protein leads to a significant accumulation of damaged proteins due to the inefficient removal of oxidized proteins, while the absence of DSS1(I) protein moderately influences oxidized protein accumulation [8]. Current research based on the functional complementation test and comparison of mutant and WT transcriptomes further confirms the differences between the two *A. thaliana DSS1* genes.

Using a semiquantitative approach, the functional complementation test demonstrated that the DSS1(I) protein of *A. thaliana* can restore the WT phenotype of the *U. maydis Δdss1* mutant. Overexpression of *A. thaliana* DSS1(I) in the *Δdss1* mutant results in significant resistance to sublethal doses of genotoxic agents. The survival rate of transformants *Δdss1* + AtDSS1(I) is 1000 times higher than that of *Δdss1*, but lower than the rate achieved in transformants *Δdss1* + UmDss1. DSS1 and BRCA2 are highly conserved proteins that interact via helix domains (HD). Literature data indicate a high degree of similarity in the HD domain, as 38% similarity is noted between *U. maydis* (Brh2) and human BRCA2, and 44% similarity between *A. thaliana* and human BRCA2 [15]. Although UmDss1 and AtDSS1 differ in their amino acid sequences, the regions responsible for interactions with partner proteins are highly conserved [15]. According to the presented data, we hypothesize that besides the differences in the amino acid sequences of the protein, DSS1(I) successfully interacts with the Brh2 complex of *U. maydis* due to evolutionary conserved domains that interact with each other. This is supported by the observation that in *A. thaliana*, DSS1(I) can bind both AtBRCA2(IV) and AtBRCA2(V) isoforms [29]. This result indicates that two variants of the *A. thaliana* DSS1 protein have different roles in recombinant DNA repair lesions. However, the absence of functional complementation of the *U. maydis Δdss1* by *A. thaliana* DSS1(V) can be attributed to the distinct structural organization of these two *A. thaliana* DSS1 proteins, which are linked to their primary amino acid sequences. Genetic redundancy in plants is the result of polyploidy, and this phenomenon allows for the accumulation of mutations, leading to the appearance of new alleles and the expansion of the gene family [30]. The evolutionary model of neofunctionalization suggests that due to gene duplication, the “ancestral” gene retains its functions while its paralog acquires new ones [31]. Given the assumption that the *AtDSS1(V)* gene is the result of gene duplication, as we discussed in our previous publication [8], and considering new results from the functional complementation tests and transcriptome analysis of the mutant lines, it is plausible to suggest that changes in protein structure during evolution contribute to the appearance of functional differences among *DSS1* genes.

To explore the potential differences in functions and uncover novel roles of the *DSS1(I)* and *DSS1(V)* genes in plant defense against oxidative stress, we analyzed the transcriptomes of *dss1* mutant seedlings and compared them not only to each other but also to the WT. Significant differences in gene expression between the CRISPR/Cas9 *dss1* mutants and WT seedlings under oxidative stress were observed. Genes showing more significant expression changes in the mutated lines compared to WT plants were subsequently classified into metabolic pathways using KEGG bioinformatics analysis.

*Dss1* mutant transcriptome analysis revealed that the biosynthesis of a broad range of protective compounds with antioxidant properties could be interrupted by their decreased gene expression due to the absence of DSS1 protein. However, there is still a high similarity between the transcriptomes of *dss1(I)* and *dss1(V)* plants regarding these metabolic pathways. Reduced expression of genes involved in the biosynthesis of secondary metabolites such as flavonoids, polyphenolic compounds, and phenylpropanoids was noted in both mutants; thus, it can be assumed that DSS1 proteins have an important role in the transcriptional regulation of the genes involved in their biosynthesis. Generally, these secondary metabolites help plants remove cytotoxic products and ROS molecules [32]. Although numerous transcriptional regulators of secondary metabolite biosynthesis have been identified and characterized, a lack of information hinders our comprehensive understanding of metabolic pathway regulation in response to environmental signals [33]. If DSS1 proteins are involved in transcriptional complexes, they could potentially play a role in regulating the synthesis of secondary metabolites through transcriptional regulation [14,23]. However, during transcriptome analysis of the mutants, it was found that some genes involved in the synthesis of phenylpropanoids have elevated expression in *dss1(I)* mutants compared to the *dss1(V)* line. This points to an unambiguous function of the DSS1(V) protein in response to oxidative stress. DSS1(V) could be a modulator of the protein network involved in initiating the expression of enzymes involved in the biosynthesis of phenylpropanoids. We assume that the absence of DSS1 proteins could make the transcriptional machinery less stable. Reducing the expression of genes that encode enzymes involved in the biosynthesis of protective compounds could render mutated plants more sensitive to oxidative stress. Additionally, we confirmed that *CYP75B* and *LDOX* are notably downregulated only in *dss1(I)* mutants. CYP75B contributes to the synthesis of plant antioxidants and thus participates in stress tolerance mechanisms, helping plants adapt to adverse conditions [34]. LDOX encodes a 2-oxoglutarate-dependent dioxygenase called anthocyanidin synthase, which converts leucoanthocyanidins to anthocyanidins, the precursors of anthocyanins. This enzyme is essential to the production of anthocyanins, which are also responsible for plant protection against UV radiation and cold stress [35,36]. In this paper, we also demonstrated that *dss1(V)* mutant plants have lower levels of AOCS6, *PR1*, and *BGLU28* transcripts when exposed to oxidative stress. Both *BGLU28* and *PR1* genes are involved in the plant defense response against biotic and abiotic stresses. BGLU28 is a beta-glucosidase enzyme that hydrolyzes glucosinolates, a class of sulfur-containing compounds that have anti-microbial and anti-herbivore activities [37]. PR1 is a pathogenesis-related protein that is induced by salicylic acid and confers resistance to fungal and bacterial pathogens [38], while AOCS6 is a MAPK substrate and a participant in ethylene biosynthesis [39]. DSS1(V) could negatively regulate various genes involved in plant protection against stress. This explains our previous results indicating that the *dss1(V)* mutant is more sensitive to stress than the *dss1(I)*. A significant difference in expression was observed in the expression profile of the *PRX70* gene among the genes involved in the process of biogenesis of secondary metabolites. It is considerably downregulated in *dss1(V)* mutants. According to different studies, this gene is involved in the removal of H_2_O_2_, the biosynthesis and degradation of lignin, the response to environmental stresses, pathogen attack, and oxidative stress [40]. However, it remains unproven whether the regulation of PRX70 enzyme transcription could be directly influenced by DSS1(V).

Transcriptome analysis revealed that the disruption of *DSS1(V)* in *dss1(V)* mutants leads to decreased expression of genes involved in the biosynthesis of plant hormones when compared to WT plants. Plant hormones also play a crucial role in plant defense against oxidative stress. Disruption of biosynthetic pathways due to DSS1 absence could cause mutant sensitivity to oxidative stress. In silico analysis of the promoter region of the *DSS1(V)* gene revealed the presence of *cis*-activating regulatory elements involved in the response to phytohormones such as jasmonates and salicylic acid [28]. In the complex and dynamic processes of transcriptional regulation in plant stress response, DSS1(V) and phytohormones could regulate each other’s expression through positive feedback mechanisms.

In comparison to WT seedlings, *dss1(I)* and *dss1(V)* mutants showed reduced expression of the genes involved in the MAPK signaling pathway under oxidative stress. MAPK has different roles in cell signal transmission, especially in the transduction of extracellular signals such as biotic and abiotic stresses, as well as in the transduction of stimuli in processes during development, differentiation, proliferation, and cell death [41]. Participants in the MAPK signaling pathway with decreased levels of transcripts also have a significant role in defense against pathogens and ROS molecules, and it is not surprising that *dss1(I)* and *dss1(V)* mutants have increased sensitivity to oxidative stress [42,43]. The downregulation of 17 genes within the MAPK cascade pathway in plants lacking the DSS1 gene may certainly disrupt plant immunity and impact their stress response. However, the specific level and mechanism by which DSS1 influences the transcriptional regulation of these genes remain unclear.

Reduced expression of certain participants in glutathione metabolism was also observed in both *dss1* mutant lines. Glutathione is a tripeptide with many vital functions, one of which is the detoxification of toxic metabolites produced under oxidative stress. In plants, the metabolism of GSH involves enzymes for the biosynthesis and degradation of GSH, as well as enzymes that utilize GSH as a substrate [44]. According to transcriptome analysis, we hypothesize that the lack of DSS1 protein also inhibits the expression of the genes involved in glutathione metabolism. It is conceivable that this inhibitory effect also contributes to the mutated *dss1* line’s inability to overcome the oxidative imbalance. On the whole, perturbations in MAPK signaling and glutathione metabolism induced by the absence of *DSS1* genes could have broader impacts on plant stress responses, cellular health, and overall adaptation to environmental fluctuations.

In contrast, both *dss1* mutant lines show increased expression of many genes involved in HR. DSS1 is one of the components of the BRCA2 complex and is a part of the HR machinery [22,29]. In this paper, several HR genes that exhibited the highest expression were considered for validation. Interestingly, the HR genes, *GIS5*, *RECG*, *RPA70*, *RECQ4A*, and *EME1B*, were upregulated in both mutants as compared to WT plants, while *BRCA1*, *CLSY3*, and *RECQ4B* showed increased expression in the transcriptome and real-time analysis, but only in the *dss1(I)* mutant. The GIS5 protein has a catalytic role within DNA polymerase δ, which participates in DNA replication and repair [45]. BRCA1, RECG, RPA70, and EME1B are involved in DNA repair [46,47,48,49], CLSY3 has ATP-dependent chromatin remodeling activity [50], while RECQ4A and RECQ4B have ATP-dependent DNA unwinding activity [51] (Table S2). DSS1(I) may represent a specific negative regulator of the transcription of these genes, considering that the absence of DSS1(I) leads to elevated levels of these genes. Although it has not been experimentally demonstrated so far, DSS1(I) may be an unusual member of the complexes along with BRCA1, CLSY3, or RECQ4B. Existing literature provides evidence that proteins belonging to the same complex could be co-regulated at the transcriptional level [52,53]. We hypothesize that the expression of DSS1 and other protein partners involved in homologous recombination may likewise be co-regulated. Consequently, if one protein is absent—as in the case of the *dss1* mutants, where DSS1 is absent—the cell may compensate for the deficiency through negative feedback. The compensatory mechanism may result in the up-regulation of other co-regulated protein partners within the complex, potentially explaining the elevated expression of the analyzed homologous recombination genes.

*Dss1(I)* mutants showed significantly elevated expression of genes that encode ABC transporters. ABC transporters are the largest family of proteins in all living organisms [45]. Compared to animals, the plant genome contains twice the number of ABC proteins. These proteins play a role in DNA repair, RNA translocation, and the active transport of numerous compounds through different types of membranes [54]. What has been established to date is that DSS1 has a potential role in proteasome regulation and RNA export. Under conditions of oxidative stress, *dss1(I)* mutant plants experience disruptions in the maintenance of cellular homeostasis. This could trigger enhanced expression of several ABC transporters to accelerate RNA transport intracellularly and eliminate toxic molecules extracellularly [54]. However, more research is needed to fully understand the molecular mechanisms and biological functions of this interplay between DSS1 proteins and ABC transporters.

The characterization of *dss1(I)* or *dss1(V)* mutant transcriptomes reveals a new potential role for DSS1 in either activating or repressing numerous genes, leading to differential gene expression in mutant lines compared to the WT. Much the same, many other intrinsically disordered proteins, DSS1 proteins have ubiquitous and diverse functions in transcriptional regulation and cell signaling [55,56]. These results and our previous findings suggest that DSS1(I) and DSS1(V) proteins play a role in various cellular processes. DSS1(I) has a role in HR, as demonstrated by the ability of AtDSS(I) to reconstitute the wild-type DNA repair phenotype of the *U. maydis Δdss1* mutant. In the absence of DSS1(I), plants showed slower developmental dynamics and the occurrence of abortive seeds. DSS1(V) provides a greater contribution to plant defense against oxidative stress and maintenance of protein homeostasis, which is evidenced by the increased sensitivity of *dss1(V)* mutant plants to oxidative stress as well as the increased accumulation of oxidized proteins compared to WT plants, attributed to impaired function of the 26S proteasome [8,28]. These findings underscore the significance of understanding the molecular mechanisms governing plant responses to oxidative stress. Understanding the role of the DSS1 protein in plant defense mechanisms could be vital for crops, especially given the prevalent factors of climate change and oxidative stress.

## 4. Materials and Methods

### 4.1. Plant Material, Cultivation, and Treatment

WT plants (Columbia-0), *dss1(I)*, and *dss1(V)* mutants of *Arabidopsis thaliana* were grown in sterile Murashige and Skoog (MS) agar medium containing 1% sucrose [57]. The homozygous mutant lines with large indels (*dss1(I)del25* and *dss1(V)ins18*) were obtained using CRISPR/Cas9 technology. The CRISPR/Cas9 gene editing technique, Arabidopsis transformation, and mutant screening have been described previously [8]. Fourteen-day-old Arabidopsis seedlings were grown for three days on agar plates containing 10 mM hydrogen peroxide. On the third day of treatment, the seedlings were harvested, immediately placed, and stored at −80 °C.

### 4.2. Ustilago Maydis Strains, Methods, and Stress Treatment

Manipulations with *Ustilago maydis*, culture methods, transformations, gene transfer, treatments, and survival after DNA damage were carried out as described [27,58]. *U. maydis’* UCM350 and Δ*dss1* strains were used for the functional complementation test. UCM350 is the nominal WT strain (*nar1-6 pan1-1 a1b1*); the Δ*dss1* strain was generated by disruption of the *UmDss1* gene in UCM350 [15,27].

The self-replicating pCM955 vector was used to express plant *AtDSS1* genes in the *U. maydis* Δ*dss1* mutant. pCM955 contains a promoter for glyceraldehyde 3-phosphate dehydrogenase (*gap*) and a positive selectable marker conferring resistance to hygromycin (the *HPH* cassette). Transformation of the *U. maydis* Δ*dss1* mutant was also carried out with the pCM1019 vector, which contains the *HPH* marker and the complementary gene for *UmDss1,* driven by the *gap* promoter.

The degree of resistance of *U. maydis* to genotoxic agents was determined semi-quantitatively. Cell counting from the overnight culture was done under a microscope (BA80, Motic, Xiamen, China) using a hemocytometer. *U. maydis* cells were grown in a-liquid rich YEPS medium (1% yeast extract, 2% sucrose, and 2% peptone) at 30 °C with agitation at 200 rpm. Spot assays were conducted by making five serial 10-fold dilutions from the initial cell suspension of 2 × 10^7^/mL. Then, 10 μL portions of each dilution were placed in order from left to right onto solid YEPS medium. Plates were incubated for three days at 30 °C for colonies to develop. To test the resistance of cells to genotoxic agents, the medium contained either 0.015% MMS or 0.0075% DEB, or an open plate was irradiated with a 254 nm germicidal UV lamp (OSRAM, Germicidal lamp, Puritec 30 W, G13 G30T8/OF, Moscow, Russia) at a dose rate of 100 J/m^2^.

### 4.3. The Functional Complementation Test of U. maydis Δdss1 Mutant

For the functional complementation test, we conducted cloning and subcloning series to analyze the genetic complementation of the *U. maydis* Δ*dss1* mutant sensitive to UV stress with the AtDSS1(I) and AtDSS1(V) proteins of *Arabidopsis thaliana*. The open reading frames of the plant *AtDSS1(I)* and *AtDSS1(V)* genes were amplified from the total cDNA of *Arabidopsis thaliana* using gene-specific primers through PCR and then subcloned into two separate pCRII plasmids (Invitrogen™, Thermo Fisher Scientific™, Waltham, MA, USA) (Table S1). cDNA was obtained from total RNA extracted from wild-type seedlings of *A. thaliana* grown on plates. The *AtDSS1(I)* cDNA sequence was amplified using the UmNdeI_F/3UTR DI_R primers, and the *AtDSS1(V)* cDNA sequence was amplified using the UmNdeI_F/3UTR DV_R primers. The recognition site for the restriction enzyme NdeI was engineered upstream of the translation start site of the *AtDSS1* gene using the primer UmNdeI_F. The PCR product was ligated into the pCRII vector using the TA Cloning™ Kit, Dual Promoter Kit (Invitrogen™, Thermo Fisher Scientific™, Waltham, MA, USA), following the manufacturer’s protocol. Electrocompetent cells MegaX DH10B T1R, transformed by the ligation mixture pCRII-*AtDSS1(I)* and pCRII-*AtDSS1(V),* were selected in a medium with 50 μg/mL kanamycin.

pCM1027, pCRII-*AtDSS1(I)*, and pCRII-*AtDSS1(V)* vectors were digested with NdeI and HindIII (Thermo Scientific Scientific™, Waltham, MA, USA). The digestion fragments were isolated by gel purification using the PureLink™ Quick Gel Extraction Kit (Invitrogen™, Thermo Fisher Scientific™, Waltham, MA, USA) according to the manufacturer’s instructions. Fragments with *AtDSS1(I)* or *AtDSS1(V)* were inserted into the linearized pCM1027 vector downstream of the *gap* promoter for expression in *U. maydis*. The transformation of the DH10B *E. coli* strain was performed with ligation products, and the transformants were selected in a medium containing 100 μg/mL ampicillin.

pCM955, pCM1027-*AtDSS1(I)*, and pCM1027-*AtDSS1(V)* vectors were digested with Acc65I and BamHI (Thermo Scientific™, Waltham, MA, USA). The fragments *gap* + *DSS1(I)* and *gap + DSS1(V)* were purified and ligated into the linearized expression pCM955 shuttle vector. The success of every cloning was validated by sequencing using universal M13 primers as well as verifying the accuracy of the gene sequences (Macrogen Europe BV, Amsterdam, The Netherlands).

### 4.4. RNA Extraction and cDNA Synthesis

For the analysis of the *AtDSS1* transcripts in *U. maydis* strains, UCM350 and Δ*dss1* strains were grown overnight, and cells were washed twice. The cells were collected, and the total RNA of each sample was extracted using the GeneJET RNA Purification Kit (Thermo Scientific Scientific™, Waltham, MA, USA) following the manufacturer’s protocol with some modifications. To increase the amount of extracted RNA, the collected cells were resuspended in yeast lysis buffer (1 M sorbitol, 0.1 M EDTA, pH 7.4) containing 20 mM dithiothreitol (DTT) and lysis enzyme from *Trichoderma harzianum* (Sigma, St. Louis, MO, USA). Suspensions were incubated for 30 min at 30 °C with agitation. Further steps were performed according to the manufacturer’s instructions. RNA purity was checked using the BioSpec-nano spectrophotometer (Shimadzu, Kyoto, Japan). Total RNA was treated with the DNA-free™ DNA Removal Kit (Invitrogen™, Thermo Fisher Scientific™, Waltham, MA, USA) to remove DNA contamination before cDNA synthesis. For cDNA synthesis, reverse transcription was performed using the RevertAid First Strand cDNA Synthesis Kit (Thermo Fisher Scientific, Waltham, MA, USA).

The examination of the transcriptome in this study involved the use of previously described 14-day-old *dss1* mutants and WT seedlings of *A. thaliana,* from which total RNA was extracted and cDNA synthesized by reverse transcription as described previously [8].

### 4.5. PCR and Gene Expression

The primer pairs used in this study are listed in Table S1. They were designed using Primer3web (accessed in August and September 2023). The primer pairs RECG and BRCA1 utilized herein were sourced from previous research [47,59]. Each 25 μL PCR reaction contained 12.5-μL of the DreamTaq Green PCR Master Mix (2×) (Thermo Fisher Scientific™, Waltham, MA, USA), 10 ng of the template, and 200nM of the primer set (Table S1). PCR reactions were incubated in the Tprofessional Thermocycler (Biometra, Jena, Germany) under the following cycling program: 5 min at 95 °C, 35 cycles at 95 °C for 30 s, at 62 °C for 30 s, at 72 °C for 30 s, and for 5 min at 72 °C. The PCR products were resolved on 2% agarose gels, stained with ethidium bromide, and visualized under UV light.

For gene expression analysis, before the SYBR Green assay, total cDNAs were diluted 1:16 with nuclease-free water. Reactions were performed in 25 µL containing 300 nM of each primer (Table S1) and Power SYBR™ Green PCR Master Mix (Applied Biosystems™, Thermo Fisher Scientific™, Waltham, MA, USA). Real-time PCR was conducted on the Magnetic Induction Cycler (Mic qPCR, Bio Molecular Systems, Queensland, Australia) under the following cycles: 10 min at 95° C, and 40 cycles at 95° C for 15 s, and 60°C for 1 min. Each PCR reaction was performed in triplicate, and no template controls were included. Amplification of PCR products was detected in real-time, and *Arabidopsis ACT2* (AT3G18780) was used as an internal normalization control. The results were analyzed with MicPCR software v2.12.7 (Bio Molecular Systems, Brisbane, Queensland, Australia) and presented as 2^−ΔCt^.

### 4.6. RNA Library Construction and Sequencing

To carry out RNA sequencing of three different biological samples (WT, *dss1(I)*, and *dss1(V)* mutants under oxidative stress), 400 ng of the isolated RNA per sample were sent to Novogene Bioinformatics Technology Co., Ltd. (Cambridge, UK) at room temperature stabilized by the DNA/RNA shield buffer (Zymo Research, Irvine, CA, USA). RNA material from each sample was collected from three biologically independent experiments [8].

Total RNA was used as input material for the RNA sample preparations. RNA purity, concentration, and quality were assessed using the RNA Nano 6000 Assay Kit of the Bioanalyzer 2100 system (Agilent Technologies, Palo Alto, CA, USA). After poly-A-based mRNA enrichment and cDNA library preparation, 150-bp pair-end RNA sequencing was conducted on the Illumina NovaSeq 6000 platform (Illumina, Inc., San Diego, CA, USA).

### 4.7. Bioinformatic Analysis

Basic data analysis with a well-annotated reference genome was carried out by Novogene (Cambridge, UK). The initial step involved processing the raw reads in FASTQ format, followed by the calculation of Q20, Q30, and the GC content for the clean reads. The raw reads were subjected to filtering, excluding any reads with adapter contamination, reads containing more than 10% of uncertain nucleotides, and low-quality reads (with a Qscore < 5 for more than 50% of the bases) using the FASTP tool (https://github.com/OpenGene/fastp). All the downstream analyses were based on clean, high-quality data.

Mapping paired-end clean reads to the *Arabidopsis thaliana* reference genome (ensemblplants_arabidopsis_thaliana_tair10_gca_000001735_1) was accomplished by HISAT2 (v2.0.5). featureCounts (v1.5.0-p3) was used to count the number of reads mapped to each gene. The FPKM (Fragments Per Kilobase of transcript per Million mapped reads) of each gene was calculated based on the length of the gene and the read count mapped to this gene.

Before analyzing differential gene expression, the read counts for each sequenced library were adjusted using the edgeR program package by applying a scaling normalization factor. Differential expression analysis of two groups was performed using the edgeR (without biological replicates) R package (v3.22.5). The *p*-values were adjusted using the Benjamini-Hochberg method to control the false discovery rate. Genes with a corrected *p*-value (padj) < 0.05 and an absolute fold change of 2 (|log2^(Fold Change)^| ≥ 1) identified by edgeR were classified as differentially expressed.

Enrichment analysis was done using the clusterProfiler (v3.8.1) R package. R package (with correction of gene length bias and threshold of corrected *p*-value < 0.05) was considered to test the statistical enrichment of differentially expressed genes in GO terms (http://www.geneontology.org/; accessed on December 2022) and KEGG pathways (http://www.genome.jp/kegg/; accessed on December 2022). The data discussed in this publication have been deposited in the NCBI Gene Expression Omnibus [60] and can be accessed through GEO Series accession number GSE264581 (https://www.ncbi.nlm.nih.gov/geo/query/acc.cgi?acc=GSE264581).

## Data Availability

The data and material that support the findings of this study are available from the corresponding author upon reasonable request.

## Supplementary Materials

The supporting information can be downloaded at: https://www.mdpi.com/article/10.3390/ijms25126291/s1.

## References

[1] Singh A., Kumar A., Yadav S., Singh I.K. (2019). Reactive oxygen species-mediated signaling during abiotic stress. Plant Gene.

[2] Demidchik V. (2015). Mechanisms of oxidative stress in plants: From classical chemistry to cell biology. Environ. Exp. Bot..

[3] Das K., Roychoudhury A. (2014). Reactive oxygen species (ROS) and response of antioxidants as ROS-scavengers during environmental stress in plants. Front. Environ. Sci..

[4] Hasanuzzaman M., Bhuyan M.B., Zulfiqar F., Raza A., Mohsin S.M., Mahmud J.A., Fujita M., Fotopoulos V. (2020). Reactive oxygen species and antioxidant defense in plants under abiotic stress: Revisiting the crucial role of a universal defense regulator. Antioxidants.

[5] Sun X., Rikkerink E.H., Jones W.T., Uversky V.N. (2013). Multifarious roles of intrinsic disorder in proteins illustrate its broad impact on plant biology. Plant Cell.

[6] Hundertmark M., Hincha D.K. (2008). LEA (Late Embryogenesis Abundant) proteins and their encoding genes in *Arabidopsis thaliana*. BMC Genom..

[7] Dominguez P.G., Frankel N., Mazuch J., Balbo I., Iusem N., Fernie A.R., Carrari F. (2013). ASR1 Mediates Glucose-Hormone cross Talk by Affecting Sugar Traficking in Tobacco Plants. Plant Physiol..

[8] Nikolić I., Samardžić J., Stevanović S., Miljuš-Đukić J., Milisavljević M., Timotijević G. (2023). CRISPR/Cas9-Targeted Disruption of Two Highly Homologous *Arabidopsis thaliana* DSS1 Genes with Roles in Development and the Oxidative Stress Response. Int. J. Mol. Sci..

[9] Paraskevopoulos K., Kriegenburg F., Tatham M.H., Rosner H.I., Medina B., Larsen I.B., Brandstrup R., Hardwick K.G., Hay R.T., Kragelund B.B. (2014). Dss1 is a 26S proteasome ubiquitin receptor. Mol. Cell.

[10] Ellisdon A.M., Dimitrova L., Hurt E., Stewart M. (2012). Structural basis for the assembly and nucleic acid binding of the TREX-2 transcription-export complex. Nat. Struct. Mol. Biol..

[11] Yang H., Jeffrey P.D., Miller J., Kinnucan E., Sun Y., Thoma N.H., Zheng N., Chen P.L., Lee W.H., Pavletich N.P. (2002). BRCA2 function in DNA binding and recombination from a BRCA2-DSS1-ssDNA structure. Science.

[12] Van Der Lee R., Buljan M., Lang B., Weatheritt R.J., Daughdrill G.W., Dunker A.K., Fuxreiter M., Gough J., Gsponer J., Jones D.T. (2014). Classification of intrinsically disordered regions and proteins. Chem. Rev..

[13] Le H.P., Ma X., Vaquero J., Brinkmeyer M., Guo F., Heyer W.-D., Liu J. (2020). DSS1 and ssDNA regulate oligomerization of BRCA2. Nucleic Acids Res..

[14] Schenstrøm S.M., Rebula C.A., Tatham M.H., Hendus-Altenburger R., Jourdain I., Hay R.T., Kragelund B.B., Hartmann-Petersen R. (2018). Expanded Interactome of the Intrinsically Disordered Protein Dss1. Cell Rep..

[15] Kojic M., Yang H., Kostrub C.F., Pavletich N.P., Holloman W.K. (2003). The BRCA2-interacting protein DSS1 is vital for DNA repair, recombination, and genome stability in Ustilago maydis. Mol. Cell.

[16] Kojic M., Kostrub C.F., Buchman A.R., Holloman W.K. (2002). BRCA2 homolog required for proficiency in DNA repair, recombination, and genome stability in Ustilago maydis. Mol. Cell.

[17] Kristensen C.N., Bystol K.M., Li B., Serrano L., Brenneman M.A. (2010). Depletion of DSS1 protein disables homologous recombinational repair in human cells. Mutat. Res..

[18] Liu J., Doty T., Gibson B., Heyer W.-D. (2010). Human BRCA2 protein promotes RAD51 filament formation on RPA-covered single-stranded DNA. Nat. Struct. Mol. Biol..

[19] Mishra A.P., Hartford S.A., Sahu S., Klarmann K., Chittela R.K., Biswas K., Jeon A.B., Martin B.K., Burkett S., Southon E. (2022). BRCA2-DSS1 interaction is dispensable for RAD51 recruitment at replication-induced and meiotic DNA double strand breaks. Nat. Commun..

[20] Zhao W., Vaithiyalingam S., San Filippo J., Maranon D.G., Jimenez-Sainz J., Fontenay G.V., Kwon Y., Leung S.G., Lu L., Jensen R.B. (2015). Promotion of BRCA2-Dependent Homologous Recombination by DSS1 via RPA Targeting and DNA Mimicry. Mol. Cell.

[21] Marston N.J., Richards W.J., Hughes D., Bertwistle D., Marshall C.J., Ashworth A. (1999). Interaction between the product of the breast cancer susceptibility gene BRCA2 and DSS1, a protein functionally conserved from yeast to mammals. Mol. Cell. Biol..

[22] Faza M.B., Kemmler S., Jimeno S., Gonzalez-Aguilera C., Aguilera A., Hurt E., Panse V.G. (2009). Sem1 is a functional component of the nuclear pore complex-associated messenger RNA export machinery. J. Cell Biol..

[23] Kragelund B.B., Schenstrom S.M., Rebula C.A., Panse V.G., Hartmann-Petersen R. (2016). DSS1/Sem1, a Multifunctional and Intrinsically Disordered Protein. Trends Biochem. Sci..

[24] Pick E., Hofmann K., Glickman M.H. (2009). PCI complexes: Beyond the proteasome, CSN, and eIF3 Troika. Mol. Cell.

[25] Reed R.G., Jobin G.W., Tomko R.J. (2022). Sem1/DSS1 accelerates ATP-dependent substrate unfolding by the proteasome through a conformation-dependent intercomplex contact. bioRxiv.

[26] Tomko R.J., Hochstrasser M. (2014). The intrinsically disordered Sem1 protein functions as a molecular tether during proteasome lid biogenesis. Mol. Cell.

[27] Kojic M., Mao N., Zhou Q., Lisby M., Holloman W.K. (2008). Compensatory role for Rad52 during recombinational repair in Ustilago maydis. Mol. Microbiol..

[28] Nikolić I.P., Nešić S.B., Samardžić J.T., Timotijević G.S. (2021). Intrinsically disordered protein AtDSS1(V) participates in plant defense response to oxidative stress. Protoplasma.

[29] Dray E., Siaud N., Dubois E., Doutriaux M.P. (2006). Interaction between Arabidopsis Brca2 and its partners Rad51, Dmc1, and Dss1. Plant Physiol..

[30] Roulin A., Auer P.L., Libault M., Schlueter J., Farmer A., May G., Stacey G., Doerge R.W., Jackson S.A. (2013). The fate of duplicated genes in a polyploid plant genome. Plant J..

[31] Panchy N., Lehti-Shiu M., Shiu S.-H. (2016). Evolution of Gene Duplication in Plants. Plant Physiol..

[32] Falcone Ferreyra M.L., Rius S.P., Casati P. (2012). Flavonoids: Biosynthesis, biological functions, and biotechnological applications. Front. Plant Sci..

[33] Liu W., Feng Y., Yu S., Fan Z., Li X., Li J., Yin H. (2021). The Flavonoid Biosynthesis Network in Plants. Int. J. Mol. Sci..

[34] Lui A.C.W., Lam P.Y., Chan K.H., Wang L., Tobimatsu Y., Lo C. (2020). Convergent recruitment of 5′-hydroxylase activities by CYP75B flavonoid B-ring hydroxylases for tricin biosynthesis in Medicago legumes. New Phytol..

[35] Li K., Zhang M., Chen H., Peng J., Jiang F., Shi X., Bai Y., Jian M., Jia Y. (2019). Anthocyanins from black peanut skin protect against UV-B induced keratinocyte cell and skin oxidative damage through activating Nrf 2 signaling. Food Funct..

[36] He Q., Ren Y., Zhao W., Li R., Zhang L. (2020). Low Temperature Promotes Anthocyanin Biosynthesis and Related Gene Expression in the Seedlings of Purple Head Chinese Cabbage (*Brassica rapa* L.). Genes.

[37] Zhang L., Kawaguchi R., Morikawa-Ichinose T., Allahham A., Kim S.-J., Maruyama-Nakashita A. (2020). Sulfur Deficiency-Induced Glucosinolate Catabolism Attributed to Two β-Glucosidases, BGLU28 and BGLU30, is Required for Plant Growth Maintenance under Sulfur Deficiency. Plant Cell Physiol..

[38] Chen N., Shao Q., Xiong Z. (2023). Isolation and characterization of a pathogenesis-related protein 1 (SlPR1) gene with induced expression in tomato (*Solanum lycopersicum*) during Ralstonia solanacearum infection. Gene.

[39] Joo S., Liu Y., Lueth A., Zhang S. (2008). MAPK phosphorylation-induced stabilization of ACS6 protein is mediated by the non-catalytic C-terminal domain, which also contains the cis-determinant for rapid degradation by the 26S proteasome pathway. Plant J..

[40] Liu Y., Cai Y., Li Y., Zhang X., Shi N., Zhao J., Yang H. (2022). Dynamic changes in the transcriptome landscape of *Arabidopsis thaliana* in response to cold stress. Front. Plant Sci..

[41] Taj G., Agarwal P., Grant M., Kumar A. (2010). MAPK machinery in plants: Recognition and response to different stresses through multiple signal transduction pathways. Plant Signal. Behav..

[42] Birkenbihl R.P., Diezel C., Somssich I.E. (2012). Arabidopsis WRKY33 is a key transcriptional regulator of hormonal and metabolic responses toward Botrytis cinerea infection. Plant Physiol..

[43] Rentel M.C., Lecourieux D., Ouaked F., Usher S.L., Petersen L., Okamoto H., Knight H., Peck S.C., Grierson C.S., Hirt H. (2004). OXI1 kinase is necessary for oxidative burst-mediated signalling in Arabidopsis. Nature.

[44] Uzilday B., Ozgur R., Sekmen A.H., Turkan I. (2017). Endoplasmic reticulum stress regulates glutathione metabolism and activities of glutathione related enzymes in Arabidopsis. Funct. Plant Biol..

[45] Iglesias F.M., Bruera N.A., Dergan-Dylon S., Marino-Buslje C., Lorenzi H., Mateos J.L., Turck F., Coupland G., Cerdán P.D. (2015). The Arabidopsis DNA polymerase δ has a role in the deposition of transcriptionally active epigenetic marks, development and flowering. PLoS Genet..

[46] Yoshida K., Miki Y. (2004). Role of BRCA1 and BRCA2 as regulators of DNA repair, transcription, and cell cycle in response to DNA damage. Cancer Sci..

[47] Wallet C., Le Ret M., Bergdoll M., Bichara M., Dietrich A., Gualberto J.M. (2015). The RECG1 DNA translocase is a key factor in recombination surveillance, repair, and segregation of the mitochondrial DNA in Arabidopsis. Plant Cell.

[48] Takashi Y., Kobayashi Y., Tanaka K., Tamura K. (2009). Arabidopsis replication protein A 70a is required for DNA damage response and telomere length homeostasis. Plant Cell Physiol..

[49] Geuting V., Kobbe D., Hartung F., Durr J., Focke M., Puchta H. (2009). Two distinct MUS81-EME1 complexes from Arabidopsis process Holliday junctions. Plant Physiol..

[50] Shang J.Y., He X.J. (2022). Chromatin-remodeling complexes: Conserved and plant-specific subunits in Arabidopsis. J. Integr. Plant Biol..

[51] Serra H., Lambing C., Griffin C.H., Topp S.D., Nageswaran D.C., Underwood C.J., Ziolkowski P.A., Séguéla-Arnaud M., Fernandes J.B., Mercier R. (2018). Massive crossover elevation via combination of HEI10 and recq4a recq4b during Arabidopsis meiosis. Proc. Natl. Acad. Sci. USA.

[52] Luo F., Liu J., Li J. (2010). Discovering conditional co-regulated protein complexes by integrating diverse data sources. BMC Syst. Biol..

[53] Siwiak M., Zielenkiewicz P. (2015). Co-regulation of translation in protein complexes. Biol. Direct.

[54] Lane T.S., Rempe C.S., Davitt J., Staton M.E., Peng Y., Soltis D.E., Melkonian M., Deyholos M., Leebens-Mack J.H., Chase M. (2016). Diversity of ABC transporter genes across the plant kingdom and their potential utility in biotechnology. BMC Biotechnol..

[55] Bondos S.E., Dunker A.K., Uversky V.N. (2021). On the roles of intrinsically disordered proteins and regions in cell communication and signaling. Cell Commun. Signal..

[56] Staby L., Bugge K., Falbe-Hansen R.G., Salladini E., Skriver K., Kragelund B.B. (2021). Connecting the αα-hubs: Same fold, disordered ligands, new functions. Cell Commun. Signal..

[57] Murashige T., Skoog F. (1962). A revised medium for rapid growth and bio assays with tobacco tissue cultures. Physiol. Plant..

[58] de Sena-Tomás C., Sutherland J.H., Milisavljevic M., Nikolic D.B., Pérez-Martín J., Kojic M., Holloman W.K. (2015). LAMMER kinase contributes to genome stability in Ustilago maydis. DNA Repair.

[59] Li J., Liang W., Li Y., Qian W. (2018). APURINIC/APYRIMIDINIC ENDONUCLEASE2 and ZINC FINGER DNA 3′-PHOSPHOESTERASE play overlapping roles in the maintenance of epigenome and genome stability. Plant Cell.

[60] Edgar R., Domrachev M., Lash A.E. (2002). Gene Expression Omnibus: NCBI gene expression and hybridization array data repository. Nucleic Acids Res..

