# Supplementary material for: Assessing Transcriptomic Responses to Oxidative Stress: Contrasting Wild-Type Arabidopsis Seedlings with dss1(I) and dss1(V) Gene Knockout Mutants
Source: Int J Mol Sci. 2024 Jun 7;25(12):6291. doi: 10.3390/ijms25126291 (PMC11203560; doi:10.3390/ijms25126291)
Supplement: Supplementary file 1 [file ijms-25-06291-s001.zip › ijms-3025796-supplementary.pdf]

Supplementary material

Assessing Transcriptomic Responses to Oxidative Stress: Contrasting Wild-Type Arabidopsis Seedlings with *dss1(I)* and *dss1(V)* Gene Knockout Mutants

Ivana Nikolić, Mira Milisavljević and Gordana Timotijević *

Group for Plant Molecular Biology, Institute of Molecular Genetics and Genetic Engineering, University of Belgrade, Vojvode Stepe 444a, 11042 Belgrade, Serbia; ivana.nikolic@imgge.bg.ac.rs (I.N.); milisavljevicm@imgge.bg.ac.rs (M.M.)

* Correspondence: timotijevic@imgge.bg.ac.rs

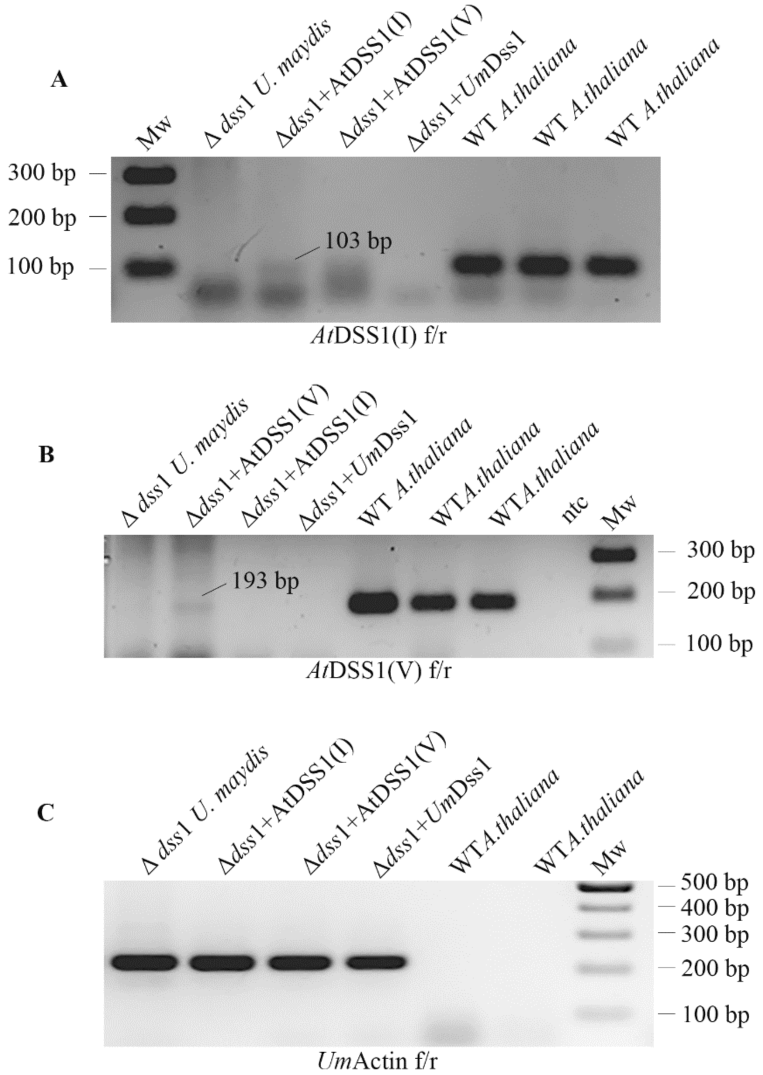

Figure S1: PCR detection of transcripts *AtDSS1(I)* and *AtDSS1(V)* isolated from *U. maydis* Δ*dss1* mutant transformants. **A** – Gel electrophoresis of PCR reactions with gene-specific primers for *A. thaliana DSS1(I)* cDNA. **B** – Gel electrophoresis of PCR reactions with gene-specific primers for *A. thaliana DSS1(V)* cDNA. **C** – Gel electrophoresis of PCR reactions with gene-specific primers for *U. maydis* actin. Δ*dss1* –*dss1* mutant of *U. maydis*; Δ*dss1*+AtDSS1(I) – Δ*dss1* mutant *U. maydis* complemented with the *AtDSS1(I)* WT sequence of *A. thaliana*; Δ*dss1*+AtDSS1(V) – Δ*dss1* mutant of *U. maydis* complemented with the *AtDSS1(V)* WT sequence of *A. thaliana*; Δ*dss1*+UmDss1 – Δ*dss1* mutant of *U. maydis* complemented with WT *UmDss1* from the genome of *U. maydis*; WT – wild strain of *A.thaliana* – positive control reaction with gene-specific primers for *AtDSS1 A. thaliana*.

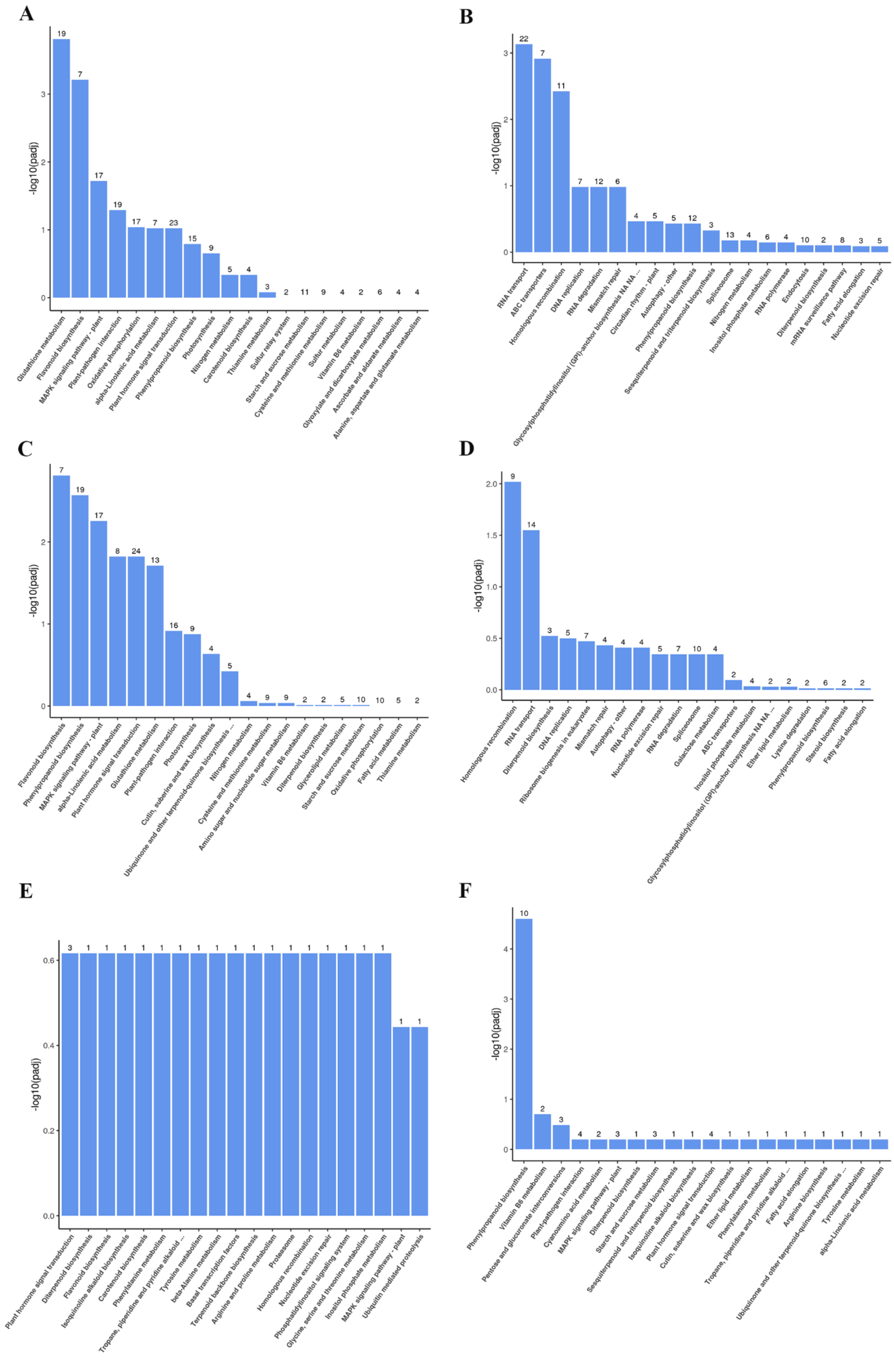
A
-log10(padj)
Glutathione metabolism
Flavonoid biosynthesis
MAPK signaling pathway - plant
Plant-pathogen interaction
Oxidative phosphorylation
alpha-Linolenic acid metabolism
Plant hormone signal transduction
Phenylpropanoid biosynthesis
Photosynthesis
Nitrogen metabolism
Carotenoid biosynthesis
Thiamine metabolism
Sulfur relay system
Starch and sucrose metabolism
Cysteine and methionine metabolism
Sulfur metabolism
Vitamin B6 metabolism
Glyoxylate and dicarboxylate metabolism
Ascorbate and aldarate metabolism
Alanine, aspartate and glutamate metabolism
B
-log10(padj)
RNA transport
ABC transporters
Homologous recombination
DNA replication
RNA degradation
Mismatch repair
Glycosylphosphatidylinositol (GPI)-anchor biosynthesis NA NA ...
Circadian rhythm - plant
Autophagy - other
Phenylpropanoid biosynthesis
Sesquiterpenoid and triterpenoid biosynthesis
Spliceosome
Nitrogen metabolism
Inositol phosphate metabolism
RNA polymerase
Endocytosis
Diterpenoid biosynthesis
mRNA surveillance pathway
Fatty acid elongation
Nucleotide excision repair
C
-log10(padj)
Flavonoid biosynthesis
Phenylpropanoid biosynthesis
MAPK signaling pathway - plant
alpha-Linolenic acid metabolism
Plant hormone signal transduction
Glutathione metabolism
Plant-pathogen interaction
Photosynthesis
Cutin, suberine and wax biosynthesis
Ubiquinone and other terpenoid-quinone biosynthesis ...
Nitrogen metabolism
Cysteine and methionine metabolism
Amino sugar and nucleotide sugar metabolism
Vitamin B6 metabolism
Diterpenoid biosynthesis
Glycerolipid metabolism
Starch and sucrose metabolism
Oxidative phosphorylation
Fatty acid metabolism
Thiamine metabolism
D
-log10(padj)
Homologous recombination
RNA transport
Diterpenoid biosynthesis
DNA replication
Ribosome biogenesis in eukaryotes
Mismatch repair
Autophagy - other
RNA polymerase
Nucleotide excision repair
RNA degradation
Spliceosome
Galactose metabolism
ABC transporters
Inositol phosphate metabolism
Glycosylphosphatidylinositol (GPI)-anchor biosynthesis NA NA ...
Ether lipid metabolism
Lysine degradation
Phenylpropanoid biosynthesis
Steroid biosynthesis
Fatty acid elongation
E
-log10(padj)
Plant hormone signal transduction
Diterpenoid biosynthesis
Flavonoid biosynthesis
Isoquinoline alkaloid biosynthesis
Carotenoid biosynthesis
Phenylalanine metabolism
Tropane, piperidine and pyridine alkaloid ...
Tyrosine metabolism
beta-Alanine metabolism
Basal transcription factors
Terpenoid backbone biosynthesis
Arginine and proline metabolism
Proteasome
Homologous recombination
Nucleotide excision repair
Phosphatidylinositol signaling system
Glycine, serine and threonine metabolism
Inositol phosphate metabolism
MAPK signaling pathway - plant
Ubiquitin mediated proteolysis
F
-log10(padj)
Phenylpropanoid biosynthesis
Vitamin B6 metabolism
Pentose and glucuronate interconversions
Plant-pathogen interaction
Cyanoamino acid metabolism
MAPK signaling pathway - plant
Diterpenoid biosynthesis
Starch and sucrose metabolism
Sesquiterpenoid and triterpenoid biosynthesis
Isoquinoline alkaloid biosynthesis
Plant hormone signal transduction
Cutin, suberine and wax biosynthesis
Ether lipid metabolism
Phenylalanine metabolism
Tropane, piperidine and pyridine alkaloid ...
Fatty acid elongation
Arginine biosynthesis
Ubiquinone and other terpenoid-quinone biosynthesis ...
Tyrosine metabolism
alpha-Linolenic acid metabolism

Figure S2: Graphical representation of the results of KEGG analysis of metabolic pathways involving proteins whose genes are down- or upregulated in seedlings treated with hydrogen peroxide. **A** – Downregulated in *dss1(I)* vs. WT; **B** – upregulated in *dss1(I)* vs. WT; **C** – downregulated in *dss1(V)* vs. WT; **D** - upregulated in *dss1(V)* vs. WT; **E** - downregulated in *dss1(I)* vs. *dss1(V)*; **F** – upregulated in *dss1(I)* vs. *dss1(V)* pathways. Descriptions of the functions of KEGG pathways are shown on the x-axis; y-axis – p-values (-log10(padj)); the order of the pathways is based on the p-value (column height). A statistically significant value of $p<0.05$ was taken for the KEGG pathways. The numbers indicate the counts of genes with an altered expression that are associated with the described pathways.

Table S1: List of primers.

Gene ID	Primer name	Primer sequence	Product size (bp)
**AT5G63960**	GIS5_F<br>GIS5_R	5′- TCCTTCGAATACCCCACCAC -3′<br>5′- CGCCGTACAAGAGAGTTTCG -3′	137
**AT2G01440**	RECG_F<br>RECG_R	5′- GGCATCAAAGAAGTCTACTCAATG -3′<br>5′- CAACGTATACTCTGCCTCCAGAC -3′	64
**AT4G21070**	BRCA1_F<br>BRCA1_R	5′- CCATGTATTTTGCAATGCGTG -3′<br>5′- TGTGGAGCACCTCGAATCTCT -3′	105
**AT5G45400**	RPA70_F<br>RPA70_R	5′- GACGCTGGGAGATATGGACA -3′<br>5′- CGGACGGGAATAAGCAGTTG -3′	171
**AT1G10930**	RECQ4A_F<br>RECQ4A_R	5′- TCATCACTCGCTCCCAAAGT -3′<br>5′- CATGGGGCTTTGGTCAACTC -3′	160
**AT1G05490**	CLSY3_F<br>CLSY3_R	5′- GGGATCAGTCTGGTAGGAGC -3′<br>5′- CTGCCCGATTCTGTAAGCAC -3′	102
**AT2G22140**	EME1B_F<br>EME1B_R	5′- TTATACGGTGTGCTGCCTCA -3′<br>5′- GCCTCGTCTACACAGTGTCT -3′	168
**AT1G60930**	RECQ4B_F<br>RECQ4B_R	5′- CCCTTCCAATGGCAAAAGCT -3′<br>5′- TTGCTCACCTTGGCTTTTCC -3′	223
**AT5G07990**	CYP75B_F<br>CYP75B_Rr	5'-AGGCGTCGCTGGTAAAATGA -3'<br>5′- ATAGCCCAGTCCACCGTACT -3′	249
**AT4G22880**	LDOX_F<br>LDOX_R	5'-TGGGGAGTGATGCATTTGATCA -3'<br>5′- ATCTTTCCAGTGGCTTGATCGT -3′	137
**AT4G11280**	AOCS6_F<br>AOCS6_R	5'- TGTAGACGAGTTTATCCGCGAG -3'<br>5'- CCGAATCAAACGTTGCTGTCTT -3'	164
**AT2G44460**	BGLU28_F<br>BGLU28_R	5'- GAACCATCGATGACCCTCTACC -3'<br>5'- AGCCCGCAACAGTTATGACATA -3'	187
**AT2G14610**	PR1_F<br>PR1_R	5'- TGTAGGTGCTCTTGTTCTTCCC -3'<br>5′- GTTGCCTCTTAGTTGTTCTGCG -3′	160
**AT5G64110**	PRX70_F<br>PRX70_R	5'- CGGACTGGGTATTACGGTAGTG- 3'<br>5'- GTCACAGCCTTGAACGAAACAA- 3'	144
**UMAG_11232**	UmActin f<br>UmActin r	5′- CTGGAAGCTGCGGGTATTCA -3′<br>5′- GAGTACTTTCGCTCGGGAGG -3′	209
**AT1G64750**	UmNdeI_F<br>3UTR DI_R	5′- TCCATATGGCGGCAGAACCG -3'<br>5′- GCAAAGGAAAAGCAGAACCG -3'	347
**AT5G45010**	UmNdeI_F<br>3UTR DV_R	5′- TCCATATGGCGGCAGAACCG -3'<br>5′- GAGAGAAGATGGTGATTATCAGAATAG-3'	431
**AT1G64750**	AtDSS1(I) f<br>AtDSS1(I) r	5′- CTGAAGTAGTAAAGATGGATCTGTTT -3'<br>5′- TGGCTAACTTCCTTCACTTCT -3'	103
**AT5G45010**	AtDSS1(V) f<br>AtDSS1(V) r	5′- AAGTGGTGAAGGTGGATCTATTC -3'<br>5′- CATTTCTTCTCACTAGCATTCTCAAG -3'	193
	M13_F<br>M13_R	5′- TCCCAGTCACGACGTCGT -3′<br>5′- GGAAACAGCTATGACCATG -3′	

Table S2: List of analyzed genes.

Gene ID	Gene name	Protein name	Function description
**AT5G63960**	*GIS5*	Gigantea suppressor 5	The catalytic subunits of DNA polymerase δ
**AT2G01440**	*RECG*	ATP-dependent DNA helicase homolog RECG	DNA repair and HR
**AT4G21070**	*BRCA1*	Breast cancer susceptibility 1 homolog	DNA repair and cell-cycle control by homologous recombination (HR)
**AT5G45400**	*RPA70*	Replication protein A 70 kDa DNA-binding subunit C	HR, DNA repair, and replication
**AT1G10930**	*RECQ4A*	ATP-dependent DNA helicase Q-like 4A	DNA repair and suppression of HR
**AT1G05490**	*CLSY3*	Chromatin remodeling 31	Gene silencing
**AT2G22140**	*EME1B*	Essential meiotic endonuclease 1b	DNA repair and HR
**AT1G60930**	*RECQ4B*	ATP-dependent DNA helicase Q-like 4B	DNA repair and HR
**AT5G07990**	*CYP75B1*	Cytochrome P450 75B1	Biosynthesis of flavonoids
**AT4G22880**	*LDOX*	Leucoanthocyanidin dioxygenase	Biosynthesis of anthocyanin and protoanthocyanidin
**AT4G11280**	*AOCS6*	1-aminocyclopropane-1-carboxylate synthase 6	Biosynthesis of ethylene
**AT2G44460**	*BGLU28*	Beta glucosidase 28	Glucosinolate catabolic process
**AT2G14610**	*PR1*	Pathogenesis-related gene 1	Defense response
**AT5G64110**	*PRX70*	Peroxidase 70	Biosynthesis and degradation of lignin
